# The Viability of Human Testis-Derived Cells on Human Serum Albumin-Based Scaffold as An Artificial Male Germ Cell Niche

**DOI:** 10.22074/ijfs.2020.6086

**Published:** 2020-07-15

**Authors:** Zahra Borzouie, Seyedhossein Hekmatimoghaddam, Ali Jebali, Behrouz Aflatoonian

**Affiliations:** 1 Stem Cell Biology Research Center, Yazd Reproductive Sciences Institute, Shahid Sadoughi University of Medical Sciences, Yazd, Iran; 2 Department of Reproductive Biology, School of Medicine, Shahid Sadoughi University of Medical Sciences, Yazd, Iran; 3 Department of Advanced Medical Sciences and Technologies, School of Paramedicine, Shahid Sadoughi University of Medical Sciences, Yazd, Iran; 4 Medical Biotechnology Research Center, Ashkezar Islamic Azad University, Ashkezar, Yazd, Iran

**Keywords:** Azoospermia, Human Serum Albumin, Scaffold, Spermatogenesis, Testis

## Abstract

Azoospermia is one of the challenging disorders affecting couples who are afflicted with infertility. Human testisderived cells (hTCs) are suitable candidates for the initiation of in-vitro spermatogenesis for these types of patients. The current study aimed to assess the proliferation of hTCs through the cell culture on the three-dimensional (3D) porous scaffolds. Cells harvested from the testicular sperm extraction (TESE) samples of the azoospermic patients were cultured on the 3D porous scaffolds containing human serum albumin (HSA)/tri calcium phosphate nanoparticles (TCP NPs) for two weeks. The proliferation/viability of the cells was assessed using the MTT assay, along with H&E histological staining method. The MTT assay showed that hTCs could stay alive on this scaffold with 50 and 66.66% viability after 7 and 14 days, respectively. Such viability was not significantly different when compared with cells grown on monolayer flask culture (P>0.05). Therefore, 3D HSA/TCP NPs scaffolds could be used for the reconstitu- tion of the artificial human somatic testicular niche for future applications in regenerative medicine for male infertility.

## Introduction

Spermatogenesis is a vital developmental phenomenon in which the production of haploid male gametes from diploid spermatogonia occurs in mammalian testes. It starts from spermatogonial stem cells (SSCs) in the seminiferous tubules with gradual differentiation toward spermatocytes, spermatids, and spermatozoa (1).

Azoospermia is defined as the lack of spermatozoa in semen, and it is one of the challenging disorders in male infertility. Approximately 1 per 200 men in any population is diagnosed as azoospermic. Although treatments, such as percutaneous epididymal sperm aspiration (PESA) and testicular sperm extraction (TESE) followed by intracytoplasmic spermatozoa injection (ICSI), are available for azoospermic patients, there is still need to improve the therapeutic approaches. As of 2004, several studies have shown that embryonic stem cells may restore the spermatogenesis and functional sperms in mouse and human, known as *in vitro* gametogenesis (IVG) (2-4).

Moreover, in parallel, several groups have demonstrated the pluripotency of germ-line stem cells (GSCs) following SSCs culture in rodents, though, there is a debate about pluripotency of GSCs in primates and humans (5).

Beside mitotic and meiotic divisions of SSCs for the production of mature spermatozoa, there are different significant factors that play roles in this process. These elements include somatic cells (such as Leydig cells, myoid cells, and Sertoli cells), extracellular matrix (ECM) components (including laminin, collagen type IV and collagen type I), as well as growth factors and hormones [including bFGF, glial cell-derived nerve factor, glial cellderived nerve factor (GDNF), and testosterone] that are capable of forming a complex microenvironment where spermatogenesis occurs (6).

By means of scaffolds, cells, and growth factors, tissue engineering has provided enormous hope and interest in academia, industry, and the public to cure various disorders (7). A recent review article by Del Vento et al. (8) indicates that tissue engineering might be helpful for the transplantation of germ cells by improving the cellular environment using scaffolds to enhance graft outcomes for prepubertal boys exposed to gonadotoxic treatments. Following our previous animal studies performed on mice (9, 10), the aim of this study was to evaluate the viability and proliferation of the cells derived from human TESE samples, which were cultivated on a novel threedimensional (3D) nano-scaffold containing human serum albumin (HSA)/tri calcium phosphate nanoparticles (TCP NPs), as examined by MTT and H&E histological staining assays. Advantages of using HSA include its low price, availability as a sterile solution, and numerous binding sites for bioactive molecules. This artificial niche could be a step forward to fertility restoration for male infertility.

TESE samples were taken after obtaining written informed consent from two non-obstructive azoospermic patients (with the ages of 27 and 36 years) who had rare immotile spermatozoa in testicular biopsies with complete spermatogenic arrest, unremarkable spermatogonia, normal Leydig cells, and normal serum hormones. The Ethics Committee of Shahid Sadoughi University of Medical Sciences in Yazd, Iran (IR.SSU.REC.1394.226). The chemicals used in this study were all purchased from Sigma-Aldrich (Poole, UK). Culture media and supplements were procured from Invitrogen (UK) unless otherwise stated. As described previously (9), in brief, 36 g of calcium nitrate [Ca (NO3)2] and 12 g of diammonium phosphate [(NH3)2 HPO4] were dissolved in 525 mL and 375 mL of distilled water (DW), respectively. Then, 25 mL of calcium nitrate was added to 25 mL of the diammonium phosphate solution, adjusted to pH=13, and kept for 6 hours at room temperature. After mild shaking, the synthesized product was washed with DW and allowed to dry. All dried TCP NPs were ball-milled for 1 hour. Then, 12.5 mg of TCP NPs were separately added to 4 mL of 500 mg/mL HSA (available as sterile injectable vials) and mixed for 1 minute. The resulting HSA/TCP NPs mixture was kept at 100°C water for 30 minutes. After the construction of solid matter, HSA/TCP NPs scaffold was frozen at -20°C, followed by the incubation at 37°C water for 30 minutes.

As explained previously (5), fresh TESE samples were placed in 2 mL of the Dulbecco’s Modified Eagle Medium (DMEM, Gibco, UK) supplemented with 5% fetal bovine serum (FBS, Gibco, UK) and transferred to the laboratory within 15 minutes. The TESE biopsies were rinsed in a Petri dish using a 19-gauge needle. TESE specimens were enzyme-dissociated overnight by the incubation in 0.1% collagenase type I in DMEM/10% FBS at 37ºC, with 5% CO_2_. Cells were subsequently recovered by aspiration and washed by centrifugation at 200 g for 3 minutes. The supernatant was discarded, and the pellet was recovered for culturing human testis-derived cells (hTCs). The obtained hTCs were incubated in flasks containing DMEM/10% FBS. Trypsin/EDTA (Gibco, UK) enzymatic method was used to passage hTCs. All cell culture experiments were performed at least in triplicate.

To sterilize the scaffolds, UV-irradiation was used for 1 hour. Following the expansion of hTCs by five passages using trypsin/EDTA, hTCs were detached from the flasks, counted, and plated on the scaffolds at a concentration of 5000 cells per well in 96-well plate culture dishes and incubated at 34°C with 5% CO_2_.

After 7 and 14 days, the cell-coated scaffolds were fixed by 4% paraformaldehyde (Sigma-Aldrich Chemie GmbH, Germany). The H&E staining method was carried out to detect arrays of hTCs within the porous scaffold.

Three cell-coated scaffolds were checked for cell proliferation/viability by the MTT [3-(4, 5-dimethyl-2-thiazolyl) -2, 5-diphenyl -2H- tetrazolium bromide] assay on days 7 and 14, and the average of 3 cultures was determined. The optical densities (ODs) at 570 nm with background subtraction at 630 nm were evaluated using an enzyme-linked immunosorbent assay (ELISA) reader (Tajhizat Sanjesh, Iran). The percentage of viability/proliferation was determined by the below formula:

Viability (%)=(OD of the test sample/OD of the control sample)×100.

For each of the 3 scaffolds, OD measurement was performed in triplicate. The statistical analysis was analyzed by the Statistical Package for the Social Sciences (SPSS) software version 22 (IBM, USA). Two-tailed bivariate (Pearson) correlations were calculated for the test and control samples based on their OD. Data are presented as mean ± SD. Differences with P<0.05 were considered statistically significant.

**Fig 1:**
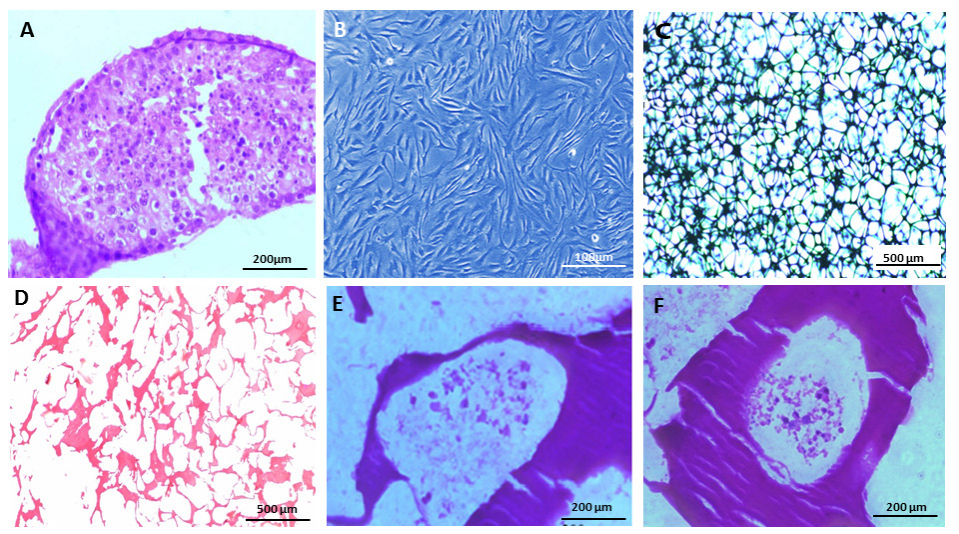
The main steps and procedures. **A.** The H&E-stained sections of TESE samples (scale bar: 200 μm), **B.** Culture of hTCs in flasks (scale bar: 100 μm), **C,** **D.** HSA/TCP NPs scaffolds (scale bar: 500 μm),** E.** Sections of 3D culture of hCs after 7 days (scale bar: 200 μm), and **F.** Sections of 3D culture of hTCs after 14 days (scale bar: 200 μm). TESE; Testicular sperm extraction, hTCs; Human testis-derived cells, HSA/TCP NPs; Human serum albumin/tricalcium phosphate nanoparticles, and 3D; Three-dimensional.

The TESE samples stained by H&E are shown in Figure 1A. hTCs were initially cultured and expanded in flasks as monolayer cell culture, and they showed mostly elongated shapes (Fig .1B). The 3D porous scaffolds containing HSA/ TCP NPs were successfully established, as described earlier. The size of pores (~10-300 μm) was checked using an inverted microscope (Fig .1C). The scaffolds were sectioned and stained (Fig .1D) before the 3D cell cultures.

Following the five passages of culture flasks, hTCs were cultured for 14 days on the 3D HSA/TCP NPs scaffolds. For the assessment of the homing and viability of the cells, cell-seeded scaffolds were sectioned and stained after 7 (Fig .1E) and 14 days (Fig .1F). H&;E staining revealed the biocompatibility of scaffolds for hTCs; nevertheless, the number of cells within the pores was dependent on the size of pores. Interestingly, there is a similarity between histological sections of TESE (Fig .1A) and hTCseeded scaffolds (Fig .1E, F); however, the latter exhibits disarrangement and possesses fewer cells.

**Fig 2:**
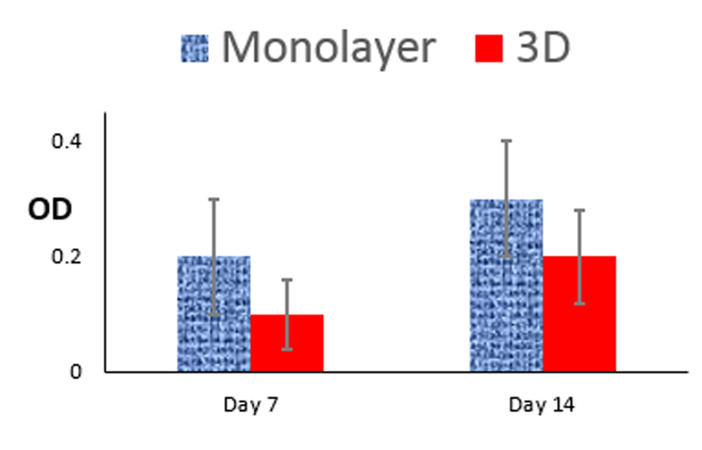
The MTT assay of hTCs cultured on monolayer versus 3D scaffold after 7 and 14 days. hTCs; Human testis-derived cells, OD; Optical density, and 3D; Three-dimensional.

The OD of monolayer cultures did not significantly (P>0.05) differ from the 3D cultures for 2 weeks (Fig .2). On day 7, the ratio of viable cells in the 3D culture was about half of that observed in the monolayer culture (0.1 ± 0.06 vs. 0.2 ± 0.10), which became 66.7% after 14 days (0.2 ± 0.08 vs. 0.3 ± 0.10). This implies the nontoxic nature of the 3D scaffold for hTCs.

Recently, successful IVG was shown in mouse embryonic stem cells for the production of sperms (11), oocytes (12), and offspring. Some potential was indicated in human embryonic stem cells but without success in achieving actual spermatozoa or oocytes (3, 4, 13). On the other hand, fertile mouse spermatozoa (14) and eggs (15) were produced using GSCs in vitro. In humans, oocytes were claimed to be produced from GSCs in women who are in the reproductive age (16), but there is still debate regarding the origin and pluripotency of GSCs and their potential for in vitro spermatogenesis (IVS) (5). One of the strategies for IVS in mouse (other than adding exogenous growth factors to the culture medium) is the transplantation of GSCs into seminiferous tubules (14). There are several challenges regarding human IVS. First of all, there are ethical and technical difficulties for the isolation of SSCs as well as the generation and expansion of putative male pluripotent GSCs. Besides, even if it becomes feasible to generate putative pluripotent GSCs from human samples in boys undergoing chemotherapy, ethical issues remain for the transplantation of GSCs in recipient testes. Tissue engineering can help to reconstitute the human somatic niche for IVS (8). In the present study, has, as an abundant source of proteins in the blood (35-50 g/L of human serum) was used for designing a homemade scaffold. HSA is a soluble globular molecule with an average half-life of 19 days. Correspondingly, it is extremely stable in a pH range of 4-9. One of the major benefits of scaffolds made from HSA is the lack of immunogenicity. Additionally, HSA produces amino acids upon the breakdown, providing nutrition for the cells in culture media. Altogether, HSA is available, cheap, biodegradable, biocompatible, and ideal candidate compound for scaffold construction (17). TCP NPs are biocompatible and biodegradable with high absorption capacity. The viability of mouse (9) and rat (10) SSCs was shown previously using HSA-based scaffolds. Our data reveal that 3D HSA/ TCP NPs scaffolds support the survival and proliferation of hTCs with 70% viability for two weeks, as compared with monolayer culture. This 3D culture system could be further studied as an artificial niche for human IVS derived from either GSCs or pluripotent stem cells.
